# Effect of Long-Term Caloric Restriction on Epigenetic Proxies of Inflammaging: CALERIE™ 2 Trial Analysis

**DOI:** 10.1093/geroni/igaf122.3753

**Published:** 2025-12-31

**Authors:** Waylon Hastings

**Affiliations:** Texas A&M University, College Station, Texas, United States

## Abstract

**Background:**

Inflammaging, the age-related increase in systemic inflammation, has received particular attention as a target for epigenetic proxies derived using machine learning approaches. Examples include InflammAge, a translational epigenetic biomarker derived from 120+ distinct epi-scores representing various inflammatory biomarkers and/or diseases, and InflClock, a measure developed to predict differences between healthy aging volunteers and patients with chronic inflammatory diseases such as rheumatoid arthritis and multiple sclerosis. Initial reports support the ability of these proxies to distinguish inflammaging phenotypes, but concerns remain about whether such measures capture meaningful change above and beyond age-related redistribution of immune cell populations. Moreover, their responsiveness to gero-protective interventions also remains untested.

**Methods:**

To address this gap, we are implementing a panel (n = 130) of inflammatory-related epi-scores in the CALERIE™ clinical trial (n = 594), including three proxies assessing global inflammaging. The overarching goal of this research proposal is to assess longitudinal concordance between epi-scores and serum biomarkers of inflammation (e.g., CRP, IL-6, TNFa), oxidative stress (e.g., isoprostanes), and immune cell distributions. Further, we leverage mixed-effect modeling to test treatment effects of the CALERIE™ intervention on these epigenetic proxies for inflammaging.

**Results:**

Preliminary analyses support associations between epigenetic proxies and some, but not all inflammatory biomarkers, and nearly all measures are associated with complete blood count differentials. Although de-noising epigenetic proxies using DNA-methylation deconvolution references increases their measurement sensitivity, intervention effect sizes remained modest to insignificant.

**Conclusions:**

Although epigenetic proxies are gaining momentum as surrogate endpoints, additional methodological refinement is necessary to achieve the rigor necessary for clinical translation.

